# Incident Stroke and Its Influencing Factors in Patients With Type 2 Diabetes Mellitus and/or Hypertension: A Prospective Cohort Study

**DOI:** 10.3389/fcvm.2022.770025

**Published:** 2022-02-09

**Authors:** Wei-Wei Chang, Shi-Zao Fei, Na Pan, Ying-Shui Yao, Yue-Long Jin

**Affiliations:** ^1^Department of Epidemiology and Health Statistics, School of Public Health, Wannan Medical College, Wuhu, China; ^2^Department of Neurology, The Second People's Hospital, Wuhu, China; ^3^The Fifth People's Hospital of Wuhu City (Wannan Rehabilitation Hospital), Wuhu, China; ^4^Anhui College of Traditional Chinese Medicine, Wuhu, China

**Keywords:** stroke, diabetes mellitus, hypertension, stroke risk factors, atherosclerosis

## Abstract

**Objective:**

To understand the incidence of stroke in patients with type 2 diabetes mellitus (T2DM) and/or hypertension (HTN), and provide a basis for the prevention of stroke in these patients.

**Methods:**

A prospective cohort study was performed for adults with T2DM and/or HTN. The follow-up period was 1 year. The incidence and recurrence rate of stroke was calculated and a multivariate Cox proportional hazard was used to analyze influencing factors of stroke occurrence and recurrence in the follow-up of patients with T2DM and/or HTN.

**Results:**

Of the 1,650 patients with T2DM and/or HTN, 1,213 patients had no history of stroke. After 1 year of follow-up, 147 new stroke cases occurred, and the incidence rate of stroke was 12.1%. Among the patients who had stroke history (413), there were 116 cases of stroke with a recurrence rate of 26.5%. Seven risk factors were independently associated with stroke occurrence among patients without stroke history, included smoking, abnormal total cholesterol abnormal low-density lipoprotein patients with comorbid T2DM with HTN, physical inactivity, carotid artery stenosis (CAS), and higher scores of National Institutes of Health Stroke Scale (NIHSS). Higher scores of NHISS and CAS were independent risk factors for the recurrence of stroke among patients with stroke history.

**Conclusions:**

Patients with T2DM and/or HTN have a higher rate of new stroke and recurrence after 1-year follow-up. Actively identifying the controllable risk factors, such as smoking and physical inactivity, will help reduce the risk of stroke and recurrence in patients with T2DM and HTN.

## Introduction

Stroke, a cerebral blood circulation disorder, causes stenosis, occlusion, or rupture of intracerebral arteries, manifesting as a one-time or permanent brain dysfunction (1, 2). The mortality rate of stroke is exceptionally high and poses a great threat to one's health (3). In addition, stroke also exerts a heavy burden on families and society, particularly, a considerable financial burden (4). According to the 2019 global burden of disease (GBD) research results (3), stroke is the second leading cause of death in the world. There were 80.1 million cases of stroke and 13.7 million new stroke cases in 2016 worldwide (3). Age-standardized mortality declined from 1990 to 2016, but the overall burden of stroke remains high (3). The costs of stroke care are rising, along with increasing burdens of disability, which provides the impetus for us to shift our research focus to effective stroke prevention measures. Reportedly, 75.2% of stroke-related deaths worldwide and 81.0% of stroke-induced disability adjusted life years (DALYs) were from developing countries (5). Stroke poses a serious threat to health in these countries and the world.

Although several studies have shown that the incidence rate and mortality of stroke are decreasing at different ages (6–8), stroke has become the leading cause of death and the leading cause of disability in China in recent years, especially in the rural areas (9, 10). Several recent studies have found that there are more than 2 million new cases each year, and the age-adjusted incidence of stroke is 246.8 per 100,000 person-years (11, 12). In addition, stroke recurrence rate is high. Several studies have found that the 1-year recurrence rate of stroke in China is 5.7–12.0%, and the disability and the fatality rate of patients with recurrence of stroke are higher than those of the first-onset patients (13–15). Compared to the European population, the onset age of stroke patients in China is younger, which increases associated DALYs (11). A nationwide population-based epidemiological survey concluded that stroke was associated with the highest DALYs of all diseases in China (10).

With the improvement of material living standards, the prevalence of type 2 diabetes mellitus (T2DM) and hypertension (HTN) are on the rise. Together with an aging population, high-risk factors (such as T2DM and HTN), and poor health management, the burden of stroke is expected to increase further (16). T2DM and HTN are the main risk factors for the onset and death from stroke in patients with cerebrovascular disease (9, 17). One of the most serious complications of T2DM is stroke, mainly ischemic stroke (18). Controlling blood pressure and blood sugar can prevent the morbidity and mortality due to stroke (19, 20). T2DM patients usually have various risk factors for cardiovascular disease, which accelerates the occurrence and development of atherosclerosis, resulting in a high incidence and disability rate of stroke in diabetic patients, threatening the quality of life of patients with T2DM.

Reportedly, burden of stroke is increasing in developing countries, because it ignores the control of vascular risk factors, such as HTN and DM (21, 22). Therefore, we must urgently and systematically identify influencing factors of stroke among people in high-risk populations, such as those with HTN and T2DM in China. Therefore, we can adopt effective preventive measures to reduce this risk. In recent years, research on risk factors related to new and recurrent stroke has gradually increased (23–26), but there are a few studies on high-risk patients with T2DM and/or HTN. Case-control studies can identify those risk factors associated with new and recurrent stroke, but cannot draw causal associations. Therefore, a prospective study to determine the risk factors for stroke occurrence and recurrence in patients with T2DM and/or HTN is urgently needed in China.

In this regard, we adopted a prospective study design and used Cox regression analysis to explore the risk factors related to stroke in T2DM and/or HTN patients, with and without a history of stroke. This study will help provide new evidence for influencing factors in stroke, and provide a new scientific basis for the adjustment of stroke prevention strategies and measures in China.

## Methods

### Study Design and Participants

This was a prospective cohort study of hospital patients. All patients with T2DM and/or HTN were recruited from the Second People's Hospital of Wuhu, Anhui Province, between September 2017 and September 2018. Inclusion criteria were as follows: (1) patients with acute stroke or stroke risk factors [diagnosis of stroke was in line with the WHO standard and was confirmed by cranial CT or magnetic resonance imaging (MRI)] (27). Patients with the following risk factors for stroke were selected: HTN, dyslipidemia, T2DM, atrial fibrillation, smoking, overweight/obese, poor exercise regimen, and family history of stroke. Having any three or more of the above eight items is defined as high-risk for stroke; (2) patients were over 18 years old; and (3) the participants voluntarily cooperated with the examination and research, including signature of the informed consent document. Exclusion criteria were as follows: (1) non-cerebrovascular disease, including patients with blood system diseases, malignant tumor diseases, and poor coordination; (2) patients or family members who failed to consent.

According to the 2017 Technical Program of Screening and Intervention Project for high-risk groups of stroke, all patients with T2DM and/or HTN enrolled during the study period were comprehensively evaluated and screened using questionnaires, routine physical examinations, imaging, and laboratory testing. The routine physical examination included blood and coagulation analysis as well as biochemical laboratory examination. Imaging, such as CT and MRI examined the head and neck blood vessels to observe whether the blood vessels were narrowed and identified the presence of plaque formation in all the patients. This study was approved by the Ethics Committee of the Second People's Hospital of Wuhu.

### Evaluation of Study Participants

Two research assistants collected the admission information of patients, including: (1) demographic information: gender, age, height, weight. Measurement of weight and height were performed by the nurses. BMI = weight (kg)/height^2^ (m^2^); (2) disease history: stroke, HTN, T2DM, and hyperlipidemia; (3) behavioral risk factors: smoking, drinking, and physical activity; (4) laboratory examination results: blood lipid, blood glucose, systolic blood pressure (SBP), diastolic blood pressure (DBP), total cholesterol (TC), triglycerides (TG), high-density lipoprotein (HDL-C), low-density lipoprotein (LDL-C), and homocysteine (HCY); (5) National Institutes of Health Stroke Scale (NIHSS) at admission; and (6) imaging testing results: carotid artery stenosis (CAS) was measured by MRI. According to the degree of CAS, the patients were classified into mild, moderate, and severe stenosis group.

### Outcome Events and Follow-Up

Date of hospital admission of a patient was considered the starting point and day 0 for follow-up time points. Patients were followed up for 6 months, and 1 year *via* telephone or face-to-face inquiries. The endpoint observed was stroke or stroke recurrence during follow-up. Stroke occurrence was defined as a new acute stroke event (including cerebral infarction, intracerebral hemorrhage, and subarachnoid hemorrhage) that occurred or reappeared during follow-up.

### Research Indicators and Criteria

Hypertension was defined as a SBP of at least 140 mmHg or a DBP of 90 mmHg or previous diagnosis of HTN. T2DM required a fasting blood sugar (FBS) ≥7.0 mmol/L or a 2-h postprandial blood glucose (PBG) ≥11.0 mmol/L or previous diagnosis of T2DM. Physical activity included medium or heavy manual workers and/or exercise with at least moderate intensity for more than half an hour and no <3 times a week. Smoking included those who currently smoke or have quit smoking at present. Defined time of smoking was an average daily consumption of ≥1 cigarette, lasting for 1 year or more. For drinking, there were three groups, any group is defined as drinking, with (1) regular drinking defined as liquor consumption ≥3 times/week, each time with ≥100 g; (2) had a history of liquor consumption and now has quit drinking; and (3) is a drinker, but rarely drinks heavily. Finally, neurological assessment was based on the NIHSS scale which included 15 items. NIHSS scale is widely used to evaluate and record the neurological deficit of patients with acute stroke (28, 29). The NIHSS score range from 0 to 42, with lower scores indicating better neurological function (30). According to NIHSS scores, the neurological deficit was classified into three categories: mild (scores from 0 to 4), moderate (scores from 5 to 14), and severe (scores from 15 to 42) (31).

### Statistical Analysis

Statistical analyses were performed using SPSS18.0 (version 18.0; SPSS, Inc.). The categorical variable data used rate or constituent ratio for descriptive analysis. The normality of quantitative data (age and BMI) was used by the Kolmogorov–Smirnov (K-S) test, and the results concluded that age and BMI did not accord with normality (*P* < 0.05). So age and BMI were expressed as medians (P_25_, P_75_) for descriptive analysis.

The basic characteristics of participants with stroke history and without stroke history were compared by using the Mann–Whitney *U*-test for continuous variables and Chi-squared (χ^2^) tests for categorical variables. The univariate and multivariate analysis of stroke risk factors was used by a Cox hazards proportion regression analysis. To increase the reliability of screening predictors, the variables with *P* < 0.1 in the univariate analysis (Mann–Whitney *U*-test and χ^2^ tests) were included in the multivariate analysis. In this study, two models (mode l and mode 2) were used to analyze the influencing factors; model 1 did not adjust the variables and the adjusting variables of mode 2 included: age, gender, smoking, drinking, physical activity, BMI, atrial fibrillation, TC, TG, LDL-C, HCY, CAS, NIHSS, and disease class. All statistical tests were bilateral and *p* < 0.05 was considered significant.

## Results

### The Basic and Clinical Characteristics of Participants

Between September 2017 and September 2018, we enrolled 1,650 subjects comprising of 917 (55.6%) men. Of all the subjects, 437 (26.5%) had stroke history and 1,213 (73.5%) had no stroke history. At enrollment, there were 854 (51.8%) HTN patients, 172 (10.4%) T2DM patients, and 624 (37.8%) patients with comorbid T2DM and HTN. The average level of BMI was 24.8 kg/m^2^, the proportion of smoking, drinking, and physical activity was 23.9, 14.7, and 12.9%, respectively. According to whether the subjects had the history of stroke, the basic and clinical characteristics of participants are presented in Table 1.

**Table 1:** The characteristics of research objects (*n* = 1,650).

**Variables**	**Total (*n* = 1,650)**	**History of stroke**		***χ*^2^/*Z***	***P***
		**No (*n* = 1,213)**	**Yes (*n* = 437)**		
Male gender, *n* (%)	917 (55.6)	666 (54.9)	251 (57.4)	0.830	0.361
Age, M (P_25_, P_75_)	68 (58,76)	66 (55,74)	73 (65,78)	9.510	<0.001
Disease class, *n* (%)					
HTN only	854 (51.8)	587 (48.4)	267 (61.1)	29.040	<0.001
T2DM only	172 (10.4)	150 (12.4)	22 (5.0)		
HTN+T2DM	624 (37.8)	476 (39.2)	148 (33.9)		
Smoking, *n* (%)	394 (23.9)	313 (25.8)	81 (18.5)	9.340	0.002
Drinking, *n* (%)	243 (14.7)	198 (16.3)	45 (10.3)	9.290	0.002
BMI, kg/m^2^, M (P_25_, P_75_)	24.8 (21.4, 28.5)	25.3 (21.4, 28.6)	24.3 (21.2, 27.9)	2.531	0.011
Physical activity, *n* (%)	213 (12.9)	180 (14.8)	33 (7.6)	15.177	<0.001
Hyperlipidemia, *n* (%)	890 (53.9)	665 (54.8)	225 (51.5)	1.438	0.230

### Incidence and Recurrence Rates of Stroke

Of the 1,650 patients with T2DM and/or HTN, 1,213 patients had no history of stroke. After 1 year of follow-up, 147 new stroke cases occurred, and the incidence rate of stroke was 12.1%. Rates among patients aged <65 and ≥65 years were 9.9 and 14.0%, respectively, and this difference between the two groups was statistically significant (χ^2^ = 4.596, *p* = 0.032; Table 2). There was no significant difference between men and women in the two age groups (*p* > 0.05). The incidence rates of stroke among patients in the T2DM, HTN, and T2DM + HTN groups were 9.3, 10.4, and 15.1%, respectively. The incidence rate of stroke was significantly different between the three groups (χ^2^ = 6.779, *p* = 0.034), but there was no significant difference between patients aged <65 and ≥ 65 years among the three disease groups (*p* > 0.05; Table 3).

**Table 2:** Incidence and recurrence rates of stroke by age groups and gender.

**Age**	**Without stroke history (*****n*** **=** **1,213)**			**Age**	**With stroke history (*****n*** **=** **437)**		
	**Males**	**Females**	**overall**		**Males**	**Females**	**overall**
<65 years	11.4%, 40 (351)	7.4%, 15 (203)	9.9%. 55 (554)	<65 years	24.6%, 16 (65)	21.4%, 9 (42)	24.5%, 25 (107)
≥65 years	16.5%, 52 (315)	11.6%, 40 (344)	14.0%, 92 (659)	≥65 years	25.8%, 48 (186)	29.9%, 43 (144)	27.6%, 91 (330)
Total	13.8%, 92 (666)	10.1%, 55 (547)	12.1%, 147 (1213)	Total	25.5%, 64 (251)	28.0%, 52 (186)	26.5%, 116 (437)
χ^2^-value	3.644	2.536	4.596	χ^2^-value	0.036	1.148	0.735
*P*-value	0.056	0.111	0.032	*P*-value	0.850	0.284	0.391

**Table 3:** Incidence and recurrence rates of stroke by age groups and disease class.

**Disease class**	**Without stroke history (*****n*** **=** **1,213)**			**Disease class**	**With stroke history (*****n*** **=** **437)**		
	**<65 years**	**≥65 years**	**overall**		**<65 years**	**≥65 years**	**overall**
HTN only	6.8%, 15 (220)	12.5%, 46 (367)	10.4%, 61 (587)	HTN only	16.9%, 10 (59)	25.0%, 52 (208)	23.2%, 62 (267)
T2DM only	9.7%, 10 (103)	8.5%, 4 (47)	9.3%, 14 (150)	T2DM only	18.2%, 2 (11)	18.2%, 2 (11)	18.2%, 4 (22)
HTN+T2DM	13.0%, 30 (231)	17.1%, 42 (245)	15.1%, 72 (476)	HTN+T2DM	35.1%, 13 (37)	33.3%, 37 (111)	33.8%, 50 (148)
Total	9.9%, 55 (554)	14.0%, 92 (659)	12.1%, 147 (1,213)	Total	23.4%, 25 (107)	27.6%, 91 (330)	26.5%, 116 (437)
χ^2^-value	4.802	3.85	6.779	χ^2^-value	4.384	3.019	6.280
*P*-value	0.091	0.146	0.034	*P*-value	0.112	0.221	0.043

Of the 1,650 patients with HTN and/or T2DM, 437 patients had stroke history. After 1 year of follow-up, there were 116 cases of stroke with a recurrence rate of 26.5%. Rates among patients aged <65 and ≥65 years were 24.5 and 27.6%, respectively, and the recurrence rate of stroke in the two groups was not statistically significant (χ^2^ = 0.735, *P* = 0.391; Table 2). There was no significant difference between men and women in the two age groups (*p* > 0.05). The recurrence rates of stroke among patients in the T2DM, HTN, and T2DM + HTN groups were 18.2, 23.2, 33.8%, respectively. The recurrence rate of stroke was significantly different between the three groups (χ^2^ = 6.280, *p* = 0.043; Table 3), but there was no significant difference between patients aged <65 and ≥65 years group among the three disease groups (*p* > 0.05; Table 3).

### The Influencing Factors for Stroke Among the Cohort

#### T2DM and/or HTN Patients Without Stroke History

Cox regression was used for univariate analysis. The specific assignments of variables are shown in Table 4. Among participants without stroke history (*n* = 1,213), female gender, ≥65 years, disease with HTN+T2DM, drinking, smoking, atrial fibrillation, abnormal TC, abnormal LDL-C, abnormal HCY, CAS, high scores of NIHSS, and physical activity were associated with stroke occurrence in Model 1 (unadjusted) analysis (Table 5).

**Table 4:** The assignment of main influencing factors of stroke incidence.

**Variables**	**Assignment**
Gender	0 = males, 1 = females
Age	0 = <65 years, 1 = ≥65 years
Hyperlipidemia	0 = no, 1 = yes
History of stroke	0 = no, 1 = yes
Disease class	0 = HTN only, 1 = T2DM only, 2 = HTN+T2DM
Smoking	0 = no, 1 = yes
Drinking	0 = no, 1 = yes
Physical activity	0 = no, 1 = yes
Atrial fibrillation	0 = no, 1 = yes
TG	0 (normal) = <2.26 mmol/L, 1 (abnormal) = ≥2.26 mmol/L
TC	0 (normal) = <6.22 mmol/L, 1 (abnormal) = ≥6.22 mmol/L
HDL-C	0 (normal) = ≥1.04 mmol/L, 1 (abnormal) = <1.04 mmol/L
LDL-C	0 (normal) = <4.14 mmol/L, 1 (abnormal) = ≥4.14 mmol/L
HCY	0 (normal) = 5–15 umol/L, 1 (abnormal) = <5 umol/L, or >15 umol/L
CAS (%)	0 (Mild) = <30, 1 (Moderate) = 30–69, 3 (Severe) = ≥70
Overweight/obese (kg/m^2^)	0 = BMI <24, 1 = BMI ≥ 24
NIHSS	0 (Mild) = <5, 1(Moderate) = 5–14, 3 (Severe) = ≥15

**Table 5:** The influencing factors of stroke in T2DM and/or HTN patients without stroke history.

**Risk factors**	***B***	**HR crude (95% CI)**	***P*-value**	***B***	HR adjusted^*^(95% CI)	***P-*value**
Gender (Male vs. female)	−0.318	0.728 (0.521–1.017)	0.062	–	–	–
Age (≥65 year vs. <65 year)	0.341	1.406 (1.007–1.9640)	0.045	0.150	1.162 (0.807–1.673)	0.419
Hyperlipidemia (Yes vs. no)	0.011	1.011 (0.731–1.400)	0.946	–	–	–
**Disease class (vs. HTN only)**						
T2DM only	−0.107	0.898 (0.502–1.605)	0.717	0.214	1.239 (0.675–2.274)	0.490
HTN+ T2DM	0.375	1.456 (1.035–2.047)	0.031	0.556	1.743 (1.216–2.499)	0.003
Smoking (Yes vs. no)	0.656	1.928 (1.386–2.681)	<0.001	0.474	1.606 (1.047–2.463)	0.030
Drinking (Yes vs. no)	0.784	2.190 (1.539–3.117)	<0.001	0.355	1.426 (0.895–2.272)	0.135
Atrial fibrillation (Yes vs. no)	0.483	1.620 (0.934–2.810)	0.086	–	–	–
TG (Abnormal vs. normal)	−0.026	0.975 (0.656–1.449)	0.899	–	–	–
TC (Abnormal vs. normal)	0.926	2.525 (1.618–3.941)	<0.001	0.509	1.664 (1.151–2.407)	0.007
HDL-C (Abnormal vs. normal)	−0.222	0.801 (0.572–1.122)	0.197	–	–	–
LDL-C (Abnormal vs. normal)	0.870	2.387 (1.458–3.908)	0.001	0.862	2.368 (1.418–3.954)	0.001
HCY (Abnormal vs. normal)	0.678	1.970 (1.217–3.189)	0.006	0.206	1.228 (0.741–2.036)	0.425
CAS (%) (vs. Mild)						
Moderate	1.294	3.649 (2.593–5.134)	<0.001	0.944	2.571 (1.773–3.728)	<0.001
Severe	2.267	9.648 (3.921–23.743)	<0.001	1.893	6.640 (2.597–16.979)	<0.001
Overweight/obese (Yes vs. no)	0.200	1.222 (0.875–1.706)	0.240	–	–	–
NIHSS (vs. mild)						
Moderate	0.619	1.857 (1.315–2.622)	<0.001	0.786	2.195 (1.419–3.397)	<0.001
Severe	1.090	2.975 (1.710–5.176)	<0.001	1.162	3.196 (1.741–5.866)	<0.001
Physical activity (Yes vs. no)	−0.983	0.374 (0.191–0.735)	0.004	−1.024	0.359 (0.177–0.728)	0.005

* *The adjustment factors were other factors besides the analysis factors, including gender, age, smoking, drinking, physical activity, disease class, atrial fibrillation, TC, LDL-C, HCY, CAS (%), NIHSS*.

The independent risk factors for incident stroke were smoking (aHR = 1.606, 95%CI: 1.047–2.463, *p* = 0.030), abnormal TC (aHR = 1.664, 95%CI: 1.151–2.407, *p* = 0.007), abnormal LDL-C (aHR = 2.368, 95%CI: 1.418–3.954, *p* = 0.001). Compared with patients in the HTN group, patients with comorbid T2DM and HTN had aHR of 1.743 (1.216–2.499), *p* = 0.003 with incident stroke. Physical activity (aHR = 0.359, 95%CI: 0.177–0.728, *p* = 0.005) was a protective factor of stroke occurrence in patients without stroke history. Compared with mild CAS, patients with moderate and severe CAS had aHR of 2.571 (1.773–3.728), *p* < 0.001 and aHR of 6.640 (2.597–16.979), *p* < 0.001 with incident stroke, respectively. Patients with NIHSS scores in the moderate and severe group had aHR of 2.195 (1.419–3.397), *p* < 0.001 and aHR of 3.196 (1.741–5.866), *p* < 0.001 with incident stroke, respectively, compared with the mild group.

#### T2DM and/or HTN With Stroke History

Among participants without stroke history (*n* = 437), disease with HTN+T2DM, drinking, smoking, atrial fibrillation, CAS, high score of NIHSS, and physical activity were associated with stroke recurrence in unadjusted analyses (*p* < 0.1; Table 6).

**Table 6:** The influencing factors of stroke in T2DM and/or HTN patients with stroke history.

**Risk factors**	***B***	**HR crude (95% CI)**	***P*-value**	***B***	HR adjusted^*^(95% CI)	***P*-value**
Gender (Male vs. female)	0.092	1.096 (0.760–1.581)	0.622	–	–	–
Age (≥65 year vs. <65 year)	0.166	1.180 (0.758–1.837)	0.463	–	–	–
Hyperlipidemia (Yes vs. no)	−0.025	0.975 (0.678–1.404)	0.893	–	–	–
Disease class (vs. HTN only)						
T2DM only	−0.245	0.783 (0.285–2.152)	0.635	0.043	1.044 (0.377–2.891)	0.934
HTN+ T2DM	0.375	1.455 (1.002–2.112)	0.049	0.334	1.396 (0.949–2.054)	0.090
Smoking (Yes vs. no)	0.515	1.674 (1.114–2.516)	0.013	0.411	1.508 (0.868–2.620)	0.145
Drinking (Yes vs. no)	0.534	1.706 (1.044–2.790)	0.033	0.081	1.085 (0.560–2.100)	0.809
Atrial fibrillation (Yes vs. no)	0.473	1.606 (0.933–2.762)	0.087	–	–	–
TG (Abnormal vs. normal)	0.283	1.327 (0.771–2.282)	0.307	–	–	–
TC (Abnormal vs. normal)	0.237	1.267 (0.517–3.105)	0.604	–	–	–
HDL-C (Abnormal vs. normal)	−0.027	0.974 (0.675–1.405)	0.887	–	–	–
LDL-C (Abnormal vs. normal)	−0.292	0.747 (0.237–2.351)	0.618	–	–	–
HCY(Abnormal vs. normal)	0.311	1.364 (0.766–2.429)	0.291	–	–	–
CAS (%) (vs.Mild)						
Moderate	1.049	2.856 (1.962–4.158)	<0.001	0.940	2.560 (1.749–3.747)	<0.001
Severe	1.761	5.818 (2.650–12.755)	<0.001	1.342	3.825 (1.669–8.771)	0.002
Overweight/obese (Yes vs. no)	0.026	1.026 (0.713–1.477)	0.890	–	–	–
NIHSS (vs. mild)						
Moderate	0.173	1.189 (0.791–1.786)	0.406	0.155	1.168 (0.771–1.768)	0.464
Severe	0.833	2.299 (1.312–4.031)	0.004	0.708	2.030 (1.147–3.594)	0.015
Physical activity (Yes vs. no)	−1.538	0.215 (0.053–0.869)	0.031	−1.344	0.261 (0.064–1.068)	0.062

* *The adjustment factors were other factors besides the analysis factors, including gender, age, smoking, drinking, physical activity, disease class, atrial fibrillation, CAS (%), NIHSS*.

In Model 2 (multivariate Cox model), only two risk factors were associated with stroke among patients with stroke history. Compared with mild CAS, patients with moderate and severe CAS had aHR of 2.560 (1.749–3.747), *p* < 0.001; and 3.825 (1.669–8.771), *p* = 0.002 with incident stroke, respectively. Patients with NIHSS scores in the moderate and severe group had aHR of 1.168 (0.771–1.768), *p* = 0.464 and 2.030 (1.147–1.068), *p* = 0.015 with incident stroke, respectively, compared with the mild group. Age, gender, hyperlipidemia, drinking, smoking, atrial fibrillation, abnormal TC, abnormal LDL-C, abnormal HCY, disease class, physical activity were not associated with stroke recurrence.

## Discussion

Stroke has the characteristics of high morbidity, high disability, high mortality, and high recurrence rate, which seriously affects the quality of life of patients and places a heavy burden on society and families (32). This study used a prospective cohort study to collect data on 1,650 patients with T2DM and/or HTN in hospital to explore the risk factors relating to new and recurring stroke, aiming to reduce the risk of relapse and disability. In this hospital-based, prospective cohort study, the new incidence and recurrence rates of stroke were 12.1 and 26.5%, respectively, in patients with T2DM and/or HTN. Seven factors, namely smoking, abnormal TC, abnormal LDL-C, patients with comorbid T2DM and HTN, CAS, NHISS, and physical inactivity, were independently associated with new stroke among T2DM and/or HTN without stroke history. Additionally, both CAS and NHISS were independently associated with stroke recurrence.

In our study, after 1 year of follow-up, patients with comorbid T2DM and HTN had the highest incidence and recurrence rate of stroke (15.1, 33.8%), followed by HTN (10.4 and 23.2%) and then T2DM (9.3 and 18.2%), which is higher than that of the general population in China (24). T2DM and HTN patients generally have various risk factors for cardiovascular disease, which accelerate the occurrence and development of atherosclerosis, resulting in a high incidence and disability of stroke in patients with diabetes and HTN (24, 33). An 8.4-year cohort study of rural Chinese HTN (>35 years old) found that the incidence of stroke in patients with HTN was 9.82% (34). Another 5.5-year cohort study indicated that the incidence of stroke among 3,315 participants aged ≥60 years with HTN was 6.21% (35). A longitudinal survey studied by Han et al. reported that the incidence of stroke in T2DM patients at the end of the 34–46 months follow-up was 5.22% (36). At present, there is no cohort study report on the incidence of stroke in patients comorbid with T2DM and HTN in the Chinese population. A recent study in a rural population in China indicated that the age-standardized rate of recurrent stroke within 1 and 5 years was 5.7 and 22.5%, respectively (13). Although the follow-up period was only 1 year, the incidence and recurrence rates of stroke in patients with T2DM and/or HTN were higher than those in the above studies, which may be related to sample selection, by including a high-risk population for stroke.

Compared with the HTN only group, patients with HTN and T2DM are associated with the increased risk of stroke. The effect of BP on stroke had a dose-response relationship, and there was no threshold (37). For each 1 mmHg increase in SBP or DBP, the risk of stroke increased by 1.05–1.40 times (38). HTN can damage the intima of blood vessels and activate the blood coagulation system, which in turn, can cause blood vessel stenosis and eventually lead to stroke (39). Several follow-up studies have shown that the risk ratio of stroke death was significantly lower in patients who adhered to the antihypertensive drug group than in those who did not (40, 41). T2DM was also an independent risk factor and patients with diabetes were 3.08 times more likely to suffer a stroke than those without (42). Diabetes can cause vascular damage by affecting the nitric oxide-dependent vasodilation function in the endogenous cardiovascular system and promoting the onset of stroke (43). A nested case-control study was conducted by Woo et al. who found that pioglitazone treatment has a significant cardiovascular preventive effect in patients with diabetes (44). HTN and T2DM are common diseases and have a high risk of attribution of stroke (25–50%) (9, 17). Both clinical trials and observational studies have shown that the use of antihypertensive and blood sugar control treatments can reduce the risk of first and recurrent stroke (19, 20). Therefore, for patients with T2DM and HTN, active control of blood sugar and blood pressure is extremely important for reducing the incidence of stroke.

The results of this study showed that the risk of stroke in smokers was 60.6% higher than that in non-smokers with T2DM and/or HTN. A meta-analysis of 14 studies concluded that smokers had an overall increased risk of stroke compared with non-smokers, with a pooled odds ratio (OR) of 1.61 (95% CI: 1.34–1.93), and there was a dose-response relationship between the number of smokers and stroke risk (45). Tobacco extract can inhibit the relaxation function of the vascular endothelium and reduce the activity of nitric oxide, leading to vascular endothelial function disorder and subsequent atherosclerosis (46). Atherosclerosis is a risk factor for stroke occurrence and recurrence. According to the census, the smoking rate of people aged 15 years and above in China was 26.6% in 2018 (47). This data suggests that more measures should be taken to control smoking rates and reduce the risk of stroke in China. In our study, the risk of stroke in patients with physical activity among T2DM and/or HTN was reduced by 64.1% compared with physical inactivity, which is consistent with previous studies (48, 49). A 10-year prospective cohort analysis by Kurth et al. showed that physical activity combined with a healthy lifestyle reduced the incidence of stroke by 55% (50). These findings suggest that lifestyle factors, such as physical inactivity, may increase the risk of first stroke. However, after adjusting for other factors, smoking and physical activity were not associated with the increased risk of recurrent stroke. This may be due to the fact that T2DM and/or HTN patients with stroke history are more concerned about their health due to stroke, and they may take the initiative to change these habits, such as smoking. Former studies have indicated that maintaining a healthy lifestyle (such as not smoking, moderate alcohol consumption, maintaining a healthy diet, normal body weight, and suitable sleep duration) can reduce or delay the occurrence of stroke (51, 52). A recent prospective cohort study has shown that maintenance of a 5-year lifestyle change scores of 2–3 or above can reduce the new risk of stroke, whereas there is no protective effect when the lifestyle score is increased from baseline ≤1–≥4 after 5-year follow-up, suggesting a healthy lifestyle needs to be improved from the baseline and maintained to a certain level to reduce the risk of stroke (53). On the contrary, a lifestyle that is too unhealthy in the early stage may not bring obvious benefits even if it improves in the later stage (53). In addition, we found that the higher levels of TC and LDL-C were associated with the increased risk of first stroke. Atherosclerosis is characterized by chronic inflammatory response. Low density lipoprotein is the basic factor for the initiation and maintenance of this chronic inflammation. Therefore, in future clinical work, attention should be paid to monitoring the blood pressure and blood lipid of patients with T2DM and/or HTN, and focusing on stroke prevention for those with abnormal indicators. At the same time, maintaining a healthy lifestyle and regular physical examination can effectively prevent the occurrence of stroke.

In this study, CAS was an independent risk factor for both the new onset and recurrent stroke. There was a significant positive correlation in our study between increase in CAS and that in the incidence of new and recurrent stroke, consistent with the results of Karlsson et al. (54). Stroke emboli mostly originate from carotid atherosclerotic plaques (55). The carotid artery is the bridge between the heart and the brain, and CAS is a key factor in stroke. Kapila et al. concluded in a study of stroke pathogenesis that 60% of hemodynamic changes were due to neck stenosis, and 70% of stroke patients had severe cervical vascular stenosis (56, 57). The main mechanism of stroke resulting from CAS involves plaque shedding to form an embolus, which leads to intracranial artery embolism and stenosis. With the aggravation of CAS, blood flow speed is accelerated, and the state of hypoperfusion of the distal vessels is more serious (58). The superposition of the two pathologies leads to an increase in the incidence of stroke. CAS is a recognized and modifiable risk factor for stroke and its recurrence (59). Reportedly, CAS was closely related to the clinical classification of stroke (60). Therefore, clinicians should use risk prediction models to screen out high-risk groups for CAS and determine the type of stroke, which plays an important role in the early detection and treatment of stroke. In addition, the present study demonstrated that a high NIHSS score was associated with the increased risk of first stroke and recurrent stroke. Song et al. indicated that NHISS could be used to predict the poor prognosis 3 months after the onset of acute ischemic stroke (61). Clinically, patients with high admission NIHSS scores should be screened for causes and early intervention, and control measures should then be adopted.

Limitations in our study should be considered. Firstly, the treatment plan for T2DM and HTN in the follow-up process was not included. Appropriate and reasonable secondary prevention treatment is important for reducing the incidence and recurrence of stroke. For T2DM and HTN patients, more attention should be paid and a comprehensive evaluation should be conducted to provide reasonable secondary prevention measures. Secondly, since the follow-up time was only 1 year, information on lifestyle changes of participants was not collected. Taking into account the long-term bad lifestyle and behavior, short-term changes may not have significant impact on related indicators, and so the role of lifestyle and behavior changes in the prevention of stroke will be further elaborated in the follow-up study. Thirdly, due to the time (1 year cohort study), only the factors affecting the new onset and recurrence of stroke were evaluated, and the long-term prognosis, including the effects of end events such as death, cannot be judged. Finally, the number of strokes included in this study was relatively small, which might have an impact on the results of multivariate analysis.

## Conclusions

In conclusion, patients with T2DM and/or HTN had a higher rate of new stroke and recurrence after 1 year of follow-up. For patients with comorbid T2DM with HTN, smoking, abnormal TC, abnormal LDL-C, physical inactivity, CAS, and a high NHISS score were independent risk factors for stroke occurrence, whereas a high NHISS score and CAS were independent risk factors for stroke recurrence. Actively identifying the above-mentioned controllable risk factors, such as smoking and physical inactivity, will help reduce the risk of stroke and recurrence in patients with T2DM and/or HTN. It is necessary to expand the sample size and increase the follow-up time to further clarify the relationship between the above factors and stroke.

## Data Availability Statement

The raw data supporting the conclusions of this article will be made available by the authors, without undue reservation.

## Ethics Statement

This study was reviewed and approved by the Ethics Committee of The Second People's Hospital of Wuhu. The patients/participants provided their written informed consent to participate in this study. Written informed consent was obtained from the individual(s) for the publication of any potentially identifiable images or data included in this article.

## Author Contributions

Y-SY and Y-LJ contributed to the study design and acquisition of data. W-WC, NP, S-ZF, and Y-LJ analyzed and interpreted the data. W-WC and Y-LJ wrote the original manuscript. All authors contributed to the article and approved the submitted version.

## Funding

This project was supported by grants from the National Natural Science Foundation of China (82003546 and 81874280), the Anhui Natural Science Foundation (1808085MH297), Anhui Province Quality Engineering (2015zjjh017), and the Fifth Batch of Talents Selected under the Special Support Plan in Anhui Province (No. T000516).

## Conflict of Interest

The authors declare that the research was conducted in the absence of any commercial or financial relationships that could be construed as a potential conflict of interest.

## Publisher's Note

All claims expressed in this article are solely those of the authors and do not necessarily represent those of their affiliated organizations, or those of the publisher, the editors and the reviewers. Any product that may be evaluated in this article, or claim that may be made by its manufacturer, is not guaranteed or endorsed by the publisher.
